# Supplementary material for: Drivers of vehicle-to-everything (V2X) adoption: A behavioral reasoning theory perspective
Source: PLoS One. 2025 Jul 3;20(7):e0327084. doi: 10.1371/journal.pone.0327084 (PMC12225831; doi:10.1371/journal.pone.0327084)
Supplement: S1 Appendix — (DOCX) [file pone.0327084.s001.docx]

**Appendix A. Measurements**

**Environmental Values** [1,2]

1. “I believe humans must respect and adapt to nature.”
2. “I believe that living in harmony with nature is essential.”
3. “I believe our actions must protect and preserve nature.”
4. “I believe protecting the environment is crucial for future generations.”

**Reasons for Adoption**

**Environmental Benefits** [3]

1. “I would use V2X technology because it can reduce vehicle emissions and improve air quality.”
2. “I would adopt V2X because it supports eco-friendly driving practices and reduces fuel consumption.”
3. “I favor V2X technology because it promotes using renewable energy sources within the transportation sector.”
4. “I believe V2X technology is beneficial because it can lead to more efficient traffic flow and reduced environmental pollution.”
5. “I support the use of V2X technology, as it aligns with my commitment to reducing the carbon footprint of transportation.”

**Economic Benefits** [3,4]

1. “V2X could reduce my travel costs through efficient routing.”
2. “I expect savings on insurance premiums with V2X safety features.”
3. “V2X’s potential to decrease traffic delays is economically beneficial.”
4. “I believe V2X technology can extend the lifespan of vehicle components.”
5. “V2X may lead to lower fuel or energy consumption by optimizing driving patterns.”

**Environmental Concerns** [5,6]

1. “I believe environmental issues have become increasingly severe in recent years.”
2. “Living in harmony with nature is essential for sustainability.”
3. “I think we must do more to conserve our dwindling natural resources.”
4. “I believe protecting the environment is a personal responsibility.”

**Technophilia** [7]

1. “My enthusiasm for technology makes V2X technology particularly appealing to me.”
2. “As a tech enthusiast, I am drawn to the innovation in EVs.”
3. “I am likely to be an early adopter of EVs.”
4. “Advances in V2X technology excite me.”

**Reasons Against Adoption**

**Risk Barrier** [8,9]

1. “I fear that using V2X communication may cause the connection to become unreliable or be lost.”
2. “I am concerned about the accuracy of information exchange in V2X systems, which could lead to errors in vehicle coordination.”
3. “I worry that the security measures for V2X technology may not be robust enough to prevent unauthorized access.”

**Cost Barrier** [10,11]

1. “The initial purchase price of V2X-enabled vehicles is too high for me.”
2. “I am concerned that the day-to-day operating costs of V2X-enabled vehicles will be higher than those of traditional vehicles.”
3. “I believe the ongoing costs of updates and maintenance for V2X technology will be higher than those for traditional vehicles.”

**Usage Barrier** [8]

1. “I am concerned about learning to use V2X features in my vehicle.”
2. “I think integrating V2X into current traffic systems seems complex.”
3. “I believe frequent updates and technical support for V2X could be inconvenient.”

**Security and Privacy Concerns** [12,13]

1. “I am concerned about data security in V2X-equipped vehicles.”
2. “I am concerned about the potential for unauthorized access to my vehicle’s V2X system.”
3. “I am concerned about protecting my privacy with V2X technology.”
4. “The risk of cyberattacks on V2X systems is a barrier for me.”
5. “I doubt that V2X communication is completely secure from hacking or surveillance.”

**Attitudes** [14–16]

1. “I am interested in utilizing V2X technology.”
2. “V2X technology is an excellent innovation.”
3. “V2X offers numerous advantages and benefits.”
4. “Purchasing a vehicle with V2X would make me happy.”

**Behavioral Intention** [17]

1. “I intend to utilize V2X technology in the future.”
2. “I plan to learn more about V2X technology to utilize it effectively.”
3. “I will recommend V2X technology to friends and family.”

**Green Behavior** [18,19]

1. “V2X technology enables me to drive in more environmentally friendly ways.”
2. “V2X systems help me take steps to act in an environmentally conscious way while driving.”
3. “V2X technology supports me in meeting eco-friendly driving expectations.”
4. “V2X technology enables me to contribute to environmental protection by driving more efficiently.”

**References**

1. Wang J, Shen M, Chu M. Why is green consumption easier said than done? Exploring the green consumption attitude-intention gap in China with behavioral reasoning theory. Clean Responsible Consum. 2021;2: 100015.

2. Zhu X, Ma Y, Kong L, Yang J. Understand consumers’ true views on new energy vehicles through behavioral reasoning and brand extension fit. Res Transp Bus Manag. 2023;49: 100974.

3. Le-Anh T, Nguyen MD, Nguyen TT, Duong KT. Energy saving intention and behavior under behavioral reasoning perspectives. Energy Effic. 2023;16: 8. doi:10.1007/s12053-023-10092-x

4. Chaveesuk S, Chaiyasoonthorn W, Kamales N, Dacko-Pikiewicz Z, Liszewski W, Khalid B. Evaluating the Determinants of Consumer Adoption of Autonomous Vehicles in Thailand—An Extended UTAUT Model. Energies. 2023;16: 855. doi:10.3390/en16020855

5. Adnan N, Nordin SM, Rahman I, Rasli AM. A new era of sustainable transport: An experimental examination on forecasting adoption behavior of EVs among Malaysian consumer. Transp Res Part A Policy Pract. 2017;103: 279–295. doi:10.1016/j.tra.2017.06.010

6. Nguyen-Phuoc DQ, Nguyen NAN, Tran PTK, Pham H-G, Oviedo-Trespalacios O. The influence of environmental concerns and psychosocial factors on electric motorbike switching intention in the global south. J Transp Geogr. 2023;113: 103705.

7. Martínez-Córcoles M, Teichmann M, Murdvee M. Assessing technophobia and technophilia: Development and validation of a questionnaire. Technol Soc. 2017;51: 183–188. doi:10.1016/j.techsoc.2017.09.007

8. Laukkanen T. Consumer adoption versus rejection decisions in seemingly similar service innovations: The case of the Internet and mobile banking. J Bus Res. 2016;69: 2432–2439. doi:10.1016/j.jbusres.2016.01.013

9. Migliore G, Wagner R, Cechella FS, Liébana-Cabanillas F. Antecedents to the Adoption of Mobile Payment in China and Italy: an Integration of UTAUT2 and Innovation Resistance Theory. Inf Syst Front. 2022;24: 2099–2122. doi:10.1007/s10796-021-10237-2

10. Marzi G, Marrucci A, Vianelli D, Ciappei C. B2B digital platform adoption by SMEs and large firms: Pathways and pitfalls. Ind Mark Manag. 2023;114: 80–93. doi:10.1016/j.indmarman.2023.08.002

11. Wong LW, Leong LY, Hew JJ, Tan GWH, Ooi KB. Time to seize the digital evolution: Adoption of blockchain in operations and supply chain management among Malaysian SMEs. Int J Inf Manage. 2020;52: 101997. doi:10.1016/j.ijinfomgt.2019.08.005

12. Faqih KMS. Factors influencing the behavioral intention to adopt a technological innovation from a developing country context: The case of mobile augmented reality games. Technol Soc. 2022;69: 101958. doi:10.1016/j.techsoc.2022.101958

13. Nikkhah HR, Grover V, Sabherwal R. Post hoc security and privacy concerns in mobile apps: the moderating roles of mobile apps’ features and providers. Inf Comput Secur. 2023. doi:10.1108/ICS-02-2023-0015

14. Ajzen I, Fishbein M. Understanding attitudes and predicting social behavior. NJ: Englewood Cliffs: Prentice-Hall; 1980.

15. Claudy MC, Garcia R, O’Driscoll A. Consumer resistance to innovation—a behavioral reasoning perspective. J Acad Mark Sci. 2015;43: 528–544. doi:10.1007/s11747-014-0399-0

16. Venkatesh V, Morris MG, Davis GB, Davis FD. User acceptance of information technology: Toward a unified view. MIS Q Manag Inf Syst. 2003;27: 425–478. doi:10.2307/30036540

17. Ajzen I. The theory of planned behavior. Organ Behav Hum Decis Process. 1991;50: 179–211. doi:10.1016/0749-5978(91)90020-T

18. Ababneh OMA. How do green HRM practices affect employees’ green behaviors? The role of employee engagement and personality attributes. J Environ Plan Manag. 2021;64: 1204–1226. doi:10.1080/09640568.2020.1814708

19. Al-Sharafi MA, Al-Emran M, Arpaci I, Iahad NA, AlQudah AA, Iranmanesh M, et al. Generation Z use of artificial intelligence products and its impact on environmental sustainability: A cross-cultural comparison. Comput Human Behav. 2023;143: 107708. doi:10.1016/j.chb.2023.107708
