# Supplementary material for: Drivers of vehicle-to-everything (V2X) adoption: A behavioral reasoning theory perspective
Source: PLoS One. 2025 Jul 3;20(7):e0327084. doi: 10.1371/journal.pone.0327084 (PMC12225831; doi:10.1371/journal.pone.0327084)
Supplement: Attachment — Submitted filename: Drivers of Vehicle to Everything .docx [file pone.0327084.s002.docx]

**Article title**：Drivers of Vehicle to Everything (V2X) Adoption: A Behavioural Reasoning Theory Perspective

**Academic comments on the article**

This article, based on Behavioral Reasoning Theory (BRT), explores the impact of environmental values, attitudes, behavioral intentions, and green behavior on V2X technology adoption, filling a gap in user behavior research in the V2X field. The research design is rigorous, using PLS-SEM to validate hypotheses, and the data collection and analysis processes are transparent. The results reveal the mechanism by which environmental values influence attitudes through dual paths of "reasons for and against," demonstrating theoretical innovation.

**Comments**

1. Although the article applies Behavioral Reasoning Theory (BRT), it does not sufficiently explain why BRT was chosen over other theories. It is recommended to supplement the unique advantages of BRT in the V2X context, such as how its dual-path analysis of "reasons for and against" better fits the complexity of technology adoption.
2. The study sample is limited to EV drivers in Turkey and was obtained through convenience sampling, which may introduce regional and selection bias. It is recommended to supplement the analysis with diversity considerations or explain how the uniqueness of the Turkish market supports the generalizability of the findings.
3. While Cronbach’s Alpha and AVE values are mentioned, the factor loadings for all constructs are not fully presented. It is recommended to supplement the complete measurement model results table or explain why some items are not displayed.
4. The study claims that PLS-SEM was chosen due to the "small sample size," but a sample of 332 already meets the minimum requirements for conventional PLS-SEM. It is recommended to revisit the rationale for method selection or supplement the explanation of how model complexity affects sample size requirements.
5. The results report β-values and significance but do not explain the practical significance of the effect sizes. It is recommended to supplement effect size metrics such as Cohen’s f² and discuss their policy or business implications.
6. The model does not include potential confounding variables, which may affect the relationship between environmental values and adoption intentions. It is recommended to address this in the limitations or expand the variable scope in future research.
7. The data statement reads "Data will be available upon request," but no clear access route or third-party hosting information is provided. It is recommended to follow PLOS ONE policies by making the data publicly available on an open platform or specifying the exact reasons for restrictions.
8. The conclusion mentions "the need for supportive policies" but does not propose specific measures. It is recommended to refine policy suggestions based on the findings.
