# Supplementary material for: Drivers of vehicle-to-everything (V2X) adoption: A behavioral reasoning theory perspective
Source: PLoS One. 2025 Jul 3;20(7):e0327084. doi: 10.1371/journal.pone.0327084 (PMC12225831; doi:10.1371/journal.pone.0327084)
Supplement: Attachment — Submitted filename: Response to Reviewers.docx [file pone.0327084.s004.docx]

**Manuscript ID:** PONE-D-25-07382

**Manuscript Title:** Drivers of Vehicle-to-Everything (V2X) Adoption: A Behavioral Reasoning Theory Perspective

**Authors’ Response to the Journal Editor and Reviewers**

The authors are very grateful to the Editor-in-Chief's efforts in essentially discussing the manuscript reviews with the reviewers and identifying further significant points for enhancements. The authors would also like to sincerely thank the reviewers for their valuable comments and careful feedback, which significantly enhanced this work and its presentation. The productive and valuable remarks enabled us to update various parts of the manuscript, as shown by the responses to each comment below. Additionally, all updated parts in the manuscript were highlighted in red to facilitate easy tracking by the editor and reviewers.

**Reviewer #1:** This article, based on Behavioral Reasoning Theory (BRT), explores the impact of environmental values, attitudes, behavioral intentions, and green behavior on V2X technology adoption, filling a gap in user behavior research in the V2X field. The research design is rigorous, using PLS-SEM to validate hypotheses, and the data collection and analysis processes are transparent. The results reveal the mechanism by which environmental values influence attitudes through dual paths of “reasons for and against,” demonstrating theoretical innovation.

**Response:** We sincerely appreciate your thoughtful review and positive feedback on our work. We are pleased that you found our research rigorous, methodologically sound, and theoretically innovative in addressing the gap in V2X user behavior studies.

**Reviewer #2:**

1. The study samples are mainly concentrated in Türkiye, which may lead to regional deviation of results, limiting the universality and applicability of the study. Suggest expanding the sample size to include respondents from different countries and cultural backgrounds to enhance the generalizability and external validity of the study.

**Response:** We sincerely appreciate your constructive feedback. You raise a valid concern regarding the regional concentration of our sample in Türkiye and its potential impact on the generalizability of our findings. In the revised manuscript (Section 5.4, “Limitations and Future Research”), we have explicitly highlighted this limitation and discussed how regional factors (e.g., infrastructure, policy incentives, or environmental awareness) might shape the observed relationships. Further, we have now added a brief justification for the sample’s relevance (e.g., Türkiye’s growing EV market and government sustainability initiatives) in the Introduction (second paragraph). Updates parts are shown in red:

**Limitations and Future Research**

While the study provides valuable insights, it is not without some limitations. Firstly, the findings of this study are based on a specific sample and context, which limits their generalizability. It is essential to recognize that attitudes and adoption factors may vary across regions, cultures, and demographics. Future work should confirm these findings in different cultural contexts, especially in regions with different levels of V2X infrastructure maturity (e.g. Europe, East Asia or North America) to assess the universality of the BRT mechanisms identified here.

2. The study collected 1289 responses, of which only 332 were from EV drivers. The sample size may not be sufficient to fully represent the entire EV driver population, leading to regional bias in the results. Suggest increasing the sample size, especially among the target population (EV drivers), to improve the robustness and reliability of statistical analysis.

**Response:** We agree that the subset of EV drivers (n=332) may not fully capture the diversity of the broader EV driver population. However, the PLS-SEM analysis with 5000 bootstrapped resamples ensures stable parameter estimates, as this exceeds the minimum sample requirements for complex models (Hair et al., 2019). The bootstrap confidence intervals confirm the reliability of the path coefficients. Still, in the revised manuscript (Limitations and Future Research), we have explicitly discussed this limitation, emphasizing the need for caution when generalizing the results to all EV drivers.

3. The use of convenience sampling and online surveys in research may introduce selection bias. It is recommended to adopt stricter random sampling methods and combine them with offline surveys to improve sample representativeness and reduce selection bias.

**Response:** Thank you for raising this important methodological consideration. We have conducted rigorous tests to ensure the robustness of our findings regarding potential biases. We performed Harman’s single-factor test, showing no dominant factor. We also conducted the full collinearity test, confirming multicollinearity and common method bias are not serious concerns.

4. Research based on cross-sectional data cannot reveal the dynamic changes of attitudes and behaviors over time. Suggest conducting longitudinal research to track the attitudes and behavioral changes of EV drivers towards V2X technology, in order to provide a deeper understanding.

**Response:** We have explicitly discussed this limitation, emphasizing the need for longitudinal designs.

**Limitations and Future Research**

…

Secondly, this study is based on cross-sectional data and provides a snapshot of attitudes and intentions at a particular moment. Future studies should prioritize larger, more diverse samples of EV drivers, including sampling stratified across regions and demographics, to validate and extend our findings. Longitudinal designs could also track the attitudes and behavioral changes in V2X adoption. Furthermore, investigating cross-cultural differences in attitudes toward V2X adoption can provide valuable insights into the role of cultural values and norms in predicting adoption decisions.

5. Although the model adopts Behavioral Reasoning Theory (BRT), it may not cover all factors that affect the adoption of V2X technology. It is suggested to consider incorporating other relevant theories (such as technology acceptance models, social impact theories, etc.) into the research framework to construct a more comprehensive theoretical model.

**Response:** We agree that integrating complementary theories could provide a more comprehensive understanding of V2X adoption.

**Limitations and Future Research**

…

Thirdly, while BRT effectively captures reasoning processes, complementary theories can take into account specific adoption factors such as habituation or social norms. Expanding the variables considered could provide a more nuanced understanding of adoption dynamics.

6. The literature review section may not cover all important research in the relevant fields. Suggest citing “Interacting multiple model-based ETUKF for efficient state estimation of connected vehicles with V2XV communication” and “Research on multi-lane energy-saving driving strategy of connected electric vehicle based on vehicle speed prediction” to expand the scope of the literature review and provide more comprehensive background support on V2X related technology.

**Response:** We expanded the scope of the literature review and cited the following relevant articles:

Pan, C., Li, Y., Wang, J., Liang, J., & Jinyama, H. (2023). Research on multi-lane energy-saving driving strategy of connected electric vehicle based on vehicle speed prediction. Green Energy and Intelligent Transportation, 2(6), 100127. https://doi.org/10.1016/j.geits.2023.100127

Wang, Y., Hu, Z., Lou, S., & Lv, C. (2023). Interacting multiple model-based ETUKF for efficient state estimation of connected vehicles with V2V communication. Green Energy and Intelligent Transportation, 2(1), 100044. <https://doi.org/10.1016/j.geits.2022.100044>

Horta, J., Siller, M., & Villarreal-Reyes, S. (2025). Cross-layer latency analysis for 5G NR in V2X communications. PLOS ONE, 20(1), e0313772. https://doi.org/10.1371/journal.pone.0313772

Souza, G. G. de, Santos, R. R. dos, & Godoy, R. B. (2025). Optimizing power grids: A valley-filling heuristic for energy-efficient electric vehicle charging. PLOS ONE, 20(1), e0316677. https://doi.org/10.1371/journal.pone.0316677

7. As for the theoretical model developed based on “behavioral reasoning theory” proposed in this paper, the experimental part lacks the comparison between this method and other SOTA methods. It is suggested to use SOTA methods in related research fields as the baseline to prove the progressiveness of the proposed method.

**Response:** In response to this comment, we have conducted systematic comparisons with established theoretical frameworks. We included the following lines to address this comment.

**Discussion**

This study aimed to develop a theoretical model to reveal the key factors that predict “electric vehicle” (EV) drivers’ adoption of V2X technology. Accordingly, the theoretical model based on “Behavioral Reasoning Theory” (BRT) is proposed and evaluated using a PLS-SEM approach. BRT offers two distinct advantages over established technology adoption models such as TAM and UTAUT. First, it uniquely accommodates both positive and negative behavioral reasoning in a unified framework, effectively addressing the attitude-behavior paradox prevalent in sustainable technology adoption. Second, the empirical results show that BRT provides significantly more explanatory power for attitudes, explaining 61% variance.

8. For the theoretical model developed based on the “behavioral reasoning theory” proposed in this paper, some other quantitative indicators should be selected or additionally involved to reflect the progressiveness of the method proposed in this paper.

**Response:** We sincerely appreciate this valuable suggestion to strengthen the methodological rigor of our study. We added standardized path coefficient effect sizes (f²) to assess substantive impact (See Table 4). We calculated model fit indices: Standardized Root Mean Square Residual (SRMR = 0.043), Normed Fit Index (NFI = 0.900).
